# Wellbeing and quality of life among parents of individuals with Fontan physiology

**DOI:** 10.1007/s11136-025-03890-6

**Published:** 2025-01-22

**Authors:** Kate H. Marshall, Yves d’Udekem, David S. Winlaw, Diana Zannino, David S. Celermajer, Karen Eagleson, Ajay J. Iyengar, Dominica Zentner, Rachael Cordina, Gary F. Sholler, Susan R. Woolfenden, Nadine A. Kasparian

**Affiliations:** 1 Heart Centre for Children, The Sydney Children’s Hospitals Network, Sydney, NSW Australia; 2 School of Clinical Medicine, University of New South Wales, Sydney, NSW Australia; 3 Division of Cardiac Surgery, Children’s National Hospital, Washington, DC USA; 4 Heart Center, Ann & Robert H. Lurie Children’s Hospital, Chicago, IL USA; 5 Clinical Epidemiology and Biostatistics Unit, Murdoch Children’s Research Institute, Melbourne, VIC Australia; 6 Sydney Medical School, The University of Sydney, Sydney, NSW Australia; 7 Department of Cardiology, Royal Prince Alfred Hospital, Sydney, NSW Australia; 8 Queensland Paediatric Cardiac Service, Queensland Children’s Hospital, Brisbane, QLD Australia; 9 Faculty of Medicine, The University of Queensland, Brisbane, QLD Australia; 10 Paediatric and Congenital Cardiac Service, Starship Children’s Hospital, Auckland, New Zealand; 11 Department of Surgery, The University of Auckland, Auckland, New Zealand; 12 Department of Cardiology, Royal Melbourne Hospital, Melbourne, VIC Australia; 13 Department of Medicine, University of Melbourne, Melbourne, VIC Australia; 14 Institute for Women, Children and their Families, Sydney Local Health District, Sydney, NSW Australia; 15 Heart and Mind Wellbeing Center, Heart Institute and Division of Behavioral Medicine and Clinical Psychology, Cincinnati Children’s Hospital Medical Center, 3333 Burnet Avenue (MLC 7039), Cincinnati, OH 45229 USA; 16 Department of Pediatrics, University of Cincinnati College of Medicine, Cincinnati, OH USA

**Keywords:** Quality of life, Pediatric congenital heart disease, Psychosocial outcomes

## Abstract

**Purpose:**

To examine global and health-related quality of life (QOL) among parents of individuals with Fontan physiology and determine associations with sociodemographic, parent and child-related health, psychological, and relational factors.

**Methods:**

Parents participating in the Australian and New Zealand Fontan Registry (ANZFR) QOL Study (*N* = 151, Parent *Mean age* = 47.9 ± 10.2 years, age range: 31.6–79.6 years, 66% women; child *Mean age* = 16.3 ± 8.8, age range: 6.9–48.7 years, 40% female) completed a series of validated measures. Health-related QOL was assessed using the PedsQL 4.0 Core Generic Scales for adults and global QOL was assessed using a visual analogue scale (0–10).

**Results:**

Most parents (81%) reported good global QOL (≥ 6), consistent with broader population trends. Nearly one-third of parents (28%) reported at-risk health-related QOL (based on total PedsQL scores) with physical functioning most affected (44%). Psychological factors, including psychological stress and sense of coherence, emerged as the strongest correlates of global and health-related QOL, explaining an additional 16 to 30% of the variance (using marginal *R*^2^). Final models explained 35 and 57% and of the variance in global and health-related QOL, respectively (marginal *R*^2^). Relational factors, including perceived social support and family functioning contributed minimally when analyzed alongside psychological variables.

**Conclusion:**

While parents of individuals with Fontan physiology report good global QOL, challenges in health-related QOL exist. We identified key psychological, sociodemographic, and health-related factors associated with parental QOL outcomes. These data may aid early identification of physical and psychosocial difficulties and guide targeted health resource allocation for this population.

**Supplementary Information:**

The online version contains supplementary material available at 10.1007/s11136-025-03890-6.

## Introduction

Caring for a child with Fontan physiology, a unique heart circulation resulting from a series of palliative surgical procedures for single-ventricle congenital heart disease (CHD), presents both joys and challenges. In Fontan physiology, venous blood from the body bypasses the heart and flows directly into the pulmonary arteries and lungs, relying on passive flow rather than a pumping chamber [1]. This circulation allows the patient to function without a sub-pulmonary ventricle, but can lead to various long-term complications, such as liver disease, protein-losing enteropathy, arrhythmias, and heart failure [2, 3]. Consequently, individuals with Fontan physiology have diverse physical, psychosocial, and neurodevelopmental needs throughout their lives [2], requiring ongoing parent and caregiver (henceforth parents) support. These challenges, often compounded by psychological and socioeconomic stressors [4, 5], can significantly affect parental wellbeing and quality of life (QOL) [6–8]. Parents of children with single-ventricle CHD report lower QOL outcomes than those with children with biventricular CHD [8, 9] and the general population [10], especially following their child’s early cardiac surgeries [11]. While greater CHD complexity is linked to lower parental health-related QOL, long-term parental outcomes are less well understood [12–14].

Sociodemographic factors, such as greater socioeconomic disadvantage [11] and financial strain [15, 16], may adversely affect health-related QOL of parents caring for a child with complex CHD. Child-related factors, including greater caregiving needs [8, 15, 17, 18], longer hospital stays [19], frequent readmissions [19], prenatal diagnosis [19] and child temperament [8, 20] may contribute to lower parental QOL outcomes. Psychological factors (e.g., post-traumatic stress [11, 19], psychological [8, 10, 19] and parenting stress [19, 21]), along with low social support [19] and family functioning difficulties [8, 19], also influence parental QOL outcomes in CHD contexts. Sense of coherence, a concept encapsulating a person’s view of life and capacity to manage stressful situations, is associated with greater wellbeing among CHD patients [22, 23] but is understudied among parents. Evidence indicates psychological and relational factors have greater impact on parental QOL than sociodemographic and clinical variables [19], yet research investigating specific associations among parents of individuals with Fontan physiology is limited, especially for fathers and parents of adults with Fontan physiology.

This study aimed to (1) investigate global and health-related QOL among parents of individuals with Fontan physiology within the Australian and New Zealand Fontan Registry (ANZFR), a bi-national network with over 1,650 Fontan patients [24], and (2) assess associations with sociodemographic, child cardiac, parental health, psychological, and relational factors. We hypothesized psychological and relational factors would account for a greater proportion of variance in QOL outcomes than sociodemographic and clinical variables among parents sampled.

## Methods

Parents were recruited via the ANZFR QOL Study [25], a cross-sectional, population-based study designed to assess the QOL and wellbeing among people with Fontan physiology and their families. Families enrolled in the ANZFR who had consented to research contact were approached from February 2016 to September 2018. Eligible participants were aged ≥ 18 years, proficient in English, without severe intellectual disability or unmanaged psychiatric illness, and their child had not undergone a heart transplantation or Fontan takedown (i.e., a surgical reversal or modification of the Fontan circulation used to alleviate severe symptoms or complications). Families were mailed study package (e.g., information sheet, consent forms), with follow-up telephone calls and emails completed as required. Paper or web-based surveys were offered based on preference. Additional recruitment efforts included posters, social media and presentations at educational events and conferences [25]. Ethics approval was obtained from the Sydney Children’s Hospitals Network (LNR/14/SCHN/554) and Royal Children’s Hospital Melbourne (HREC REF#35067A) Human Research Ethics Committees.

Measure selection and analyses were guided by the revised Wilson and Cleary model of health-related QOL [26, 27], as outlined in the published protocol [25]. Health-related QOL was defined as a multidimensional construct influenced by an individual’s health condition, encompassing physical, psychological, social, and occupational functioning [26, 27]. The 23-item Pediatric Quality of Life Inventory 4.0 (PedsQL) Generic Core Scales [28, 29], adapted and validated for adults [30], were used to evaluate physical (Cronbach’s alpha in the present sample, α = 0.87), emotional (α = 0.84), social (α = 0.81), and occupational (α = 0.84) functioning. Participants rated items on a five-point scale, with scores reversed and scaled to 0–100, where higher scores indicate better health-related QOL. Mean scores for each subscale and a combined psychosocial functioning summary score (α = 0.90) were calculated. One standard deviation (SD) below the normative mean indicated ‘at-risk’ status [28, 30]. The PedsQL Generic Core Scales [30] demonstrated high internal consistency in this study (total PedsQL score α = 0.92). Global QOL, encompassing subjective wellbeing and life satisfaction (including health-related factors) [31], over the past month was measured using one self-report item (i.e., *“When you think about all aspects of your life over the past month, what has this been like for you?”*), with responses indicated on a 10 cm visual analogue scale from 0 (‘Worst possible’) to 10 (‘Best possible’).

Sociodemographic factors including parent age, sex, birth country, education, employment, primary language, marital status, number of children, family structure, weekly household income (relative to the Australian [32] or New Zealand [33] average), and financial stress (0 ‘Not at all worried’ to 4 ‘Extremely worried’) were self-reported. Geographic remoteness was classified using Australian [34] and New Zealand [35] standards and grouped as ‘Metropolitan or urban’ and ‘Regional or rural’. Health literacy was assessed using the Brief Health Literacy Screen (BHLS; 2 items) [36]. Parent health characteristics included self-reported comorbidities and medication use. Child cardiac-related characteristics were extracted from the ANZFR, including primary cardiac diagnosis, presence of a syndrome or extra-cardiac congenital anomaly, age at Fontan procedure, Fontan type, time since Fontan, post-operative complications (e.g., arrhythmias, stroke), current New York Heart Association class (higher values indicating poorer cardiac function), ventricular function, and AV valve regurgitation at most recent follow-up. Parents reported on their child’s comorbidities, hospital admissions, upcoming procedures, cardiology visits, medication adherence, and dietary and exercise restrictions. Perceived severity of their child’s heart condition was rated from 0 (‘Not at all serious’) to 4 (‘Extremely serious’). Parent-proxy reported child health-related QOL was assessed using the relevant PedsQL Generic Core Scales, as previously described [28, 29].

Parent psychological factors included sense of coherence (α = 0.88) [37], and symptoms of depression (α = 0.92), anxiety (α = 0.82), stress (α = 0.87; Depression, Anxiety, and Stress Scales; DASS-21) [38], and traumatic stress (Impact of Events Scale Revised; IES-R; α = 0.94) [39]. Relational factors included attachment style (Attachment Style Questionnaire Short Form [ASQ-SF]; 29 items; avoidance, α = 0.85 and anxiety α = 0.88) [40], perceived social support (Multidimensional Scale of Perceived Social Support [MSPSS]; 12 items; α = 0.96) [41], family functioning (General Functioning subscale of the Family Assessment Device [GF-12]; 12 items; α = 0.90) [42], parental reflective functioning (Parental Reflective Functioning Questionnaire [PRFQ]; 18-items; pre-mentalizing modes, α = 0.99, certainty of mental states, α = 0.92, interest and curiosity in mental states, α = 0.92) [43], and family impact of childhood chronic illness (Impact on Family Scale [IFS]; 15 items; α = 0.91) [44]. Access to and uptake of emotional support from health professionals was assessed using nine self-report items.

### Statistical analysis

Continuous variables were summarized using means (M) and SDs or medians and interquartile ranges (IQR), while categorical variables used frequencies. Differences between participants and non-participants were examined using Pearson chi-square or *t*-tests, as appropriate. Frequencies of PedsQL scores > 1 SD below the available normative means [30], indicative of at-risk status, were calculated according to the PedsQL scoring guide; however, formal statistical comparisons of parental PedsQL scores to a normative mean were not carried out due to the lack of a suitable normative sample (aged ≥ 30 years). Frequencies of DASS-21 scores were categorized by established severity levels [38], and one-sample *t*-tests compared DASS-21 scores [45] with normative data. Independent samples *t*-tests or Mann–Whitney *U* tests compared global QOL, PedsQL and DASS-21 scores between mothers and fathers, where appropriate.

Primary outcomes (total PedsQL and global QOL scores) were modelled using regression analysis, with generalized estimating equations (GEE) using an exchangeable correlation structure [46], to account for familial clustering [47]. Analytic decisions were informed by theory [26] and univariable analyses (predictors *p* < 0.10 eligible; Supplementary Table 1), with age, sex, and education included as covariates. Due to multicollinearity (Pearson correlation, *r* ≥ 0.80) among DASS-21 subscales, only the Stress subscale was retained. Similarly, sense of coherence was selected over attachment anxiety. Model fit was evaluated using the quasi-likelihood under the independence model criterion (QIC) [48], with lower QIC values indicating better fit, and marginal *R*^2^ calculated to assess variation in the dependent variable [49]. Two models were sequentially refined across five blocks: (1) sociodemographic, (2) parental health, (3) child cardiac factors, (4) parent psychological, and (5) relational factors, adjusting until the QIC no longer improved [46]. Residual plots assessed between-cluster heteroscedasticity and QIC comparisons with alternative correlation structures were examined to confirm the suitability of the exchangeable structure [46]. Multivariable analyses included only complete cases, supported by Little’s Missing at Complete Random (MCAR) test [50] (*p* = 0.35), suggesting data missingness was completely at random for variables with ≥ 10% missing. A *p* < 0.05 was deemed statistically significant, and 95% confidence intervals (CIs) were reported. Analyses utilized IBM SPSS Statistics Version 27.0 [51] for data management, *R* Version 2023.12.1 [52] for group comparisons and regression analyses [53, 54], and Plotly [55] in Python (v5.19.0) [56] for figures.

## Results

### Participant characteristics

Among 597 eligible and contactable families, 151 parents (99 mothers, 52 fathers) from 110 families (54% from same family) completed the questionnaire, yielding an 18% response rate from those enrolled in the ANZFR (Supplementary Fig. 1). No differences between participating and non-participating families were found for child age at Fontan operation (*p* = 0.25), hypoplastic left heart syndrome (HLHS) diagnosis (*p* = 0.16), presence of a syndrome or extra-cardiac congenital anomaly (*p* = 0.29), or residential area (*p* = 0.15); however, parents of younger children and children who more recently underwent the Fontan operation were more likely to participate (*p* < 0.001 for both).

Mean age of participating parents was 47.9 ± 10.2 years (Range: 31.6 to 79.6 years; Table 1). Most were Australian-born (66%), primarily English-speaking (94%), and married or in a relationship (90%). Parents had an average of 2.8 ± 1.0 children, with 38% of Fontan patients being the first-born or only child. Over half (52%) held a university degree, 75% were employed, most resided in urban areas (56%), and reported a household income above the national average (56%). Average age of participants’ child with Fontan physiology was 16.3 ± 8.8 years (Range: 6.9–48.7 years), 40% were assigned female sex at birth, and 62% were diagnosed prenatally. Commonest CHD diagnoses included double inlet left ventricle and double outlet right ventricle (15% each), followed by tricuspid atresia and HLHS (15% each). Mean age at Fontan operation was 5.1 ± 2.8 years, occurring on average 11.5 ± 8.2 years ago. Most children (75%) underwent an extracardiac conduit procedure.

**Table 1:** Sociodemographic, parent physical and child cardiac health characteristics of parents of individuals with Fontan physiology

Variable	Mothers (*n* = 99)	Fathers (*n* = 52)	Total Sample (*N* = 151)
*Sociodemographic characteristics*			
Mean age at assessment, years	46.8 ± 9.7	50.0 ± 10.8	47.90 ± 10.2
Country of birth			
Australia	71 (71%)	28 (54%)	96 (66%)
New Zealand	10 (10%)	9 (17%)	19 (13%)
Other	17 (17%)	14 (27%)	31 (21%)
Language primarily spoken at home			
English	95 (95%)	47 (90%)	142 (94%)
Other	2 (2%)	4 (8%)	6 (4%)
Marital status			
Married or partnered	86 (87%)	48 (92%)	132 (90%)
Not married	8 (8%)	3 (6%)	11 (7%)
Number of children	2.7 ± 1.0	2.98 ± 1.1	2.78 ± 1.0
Fontan patient first-born or only child, % yes	37 (37%)	17 (33%)	54 (38%)
Educational attainment			
University degree	46 (47%)	24 (46%)	79 (52%)
No university degree	52 (53%)	27 (52%)	70 (46%)
Employment status			
Employed (e.g., full-, part-time, casual)	70 (36%)	43 (83%)	113 (75%)
Unemployed (incl. job-seeking, disability pension)	5 (5%)	2 (4%)	7 (5%)
Unemployed, not looking for work (e.g., retired, carer)	20 (20%)	7 (14%)	27 (18%)
Employed hours per week, hours	28.1 ± 13.0	44.2 ± 14.2	35.3 ± 15.2
Gross weekly household income			
Below national average	29 (29%)	14 (27%)	43 (29%)
Above national average	57 (58%)	28 (54%)	85 (56%)
Perceived financial stress, scale 0–4	1.2 ± 1.1	1.1 ± 0.9	1.1 ± 1.0
Residential location			
Metropolitan or urban	57 (58%)	28 (54%)	85 (56%)
Regional or rural	27 (27%)	13 (25%)	40 (27%)
Health literacy, scale 0–8	7.4 ± 0.9	7.0 ± 1.2	7.3 ± 1.0
*Parental health characteristics*			
Acute or chronic health condition, % yes	40 (40%)	23 (44%)	63 (42%)
Medication use, % yes	45 (46%)	22 (42%)	67 (44%)
*Child cardiac characteristics*			
Child age, years	16.4 ± 8.6	16.3 ± 9.1	16.3 ± 8.8
Child sex, % female	43 (43%)	18 (35%)	61 (40%)
Prenatal diagnosis, % yes	60 (61%)	33 (64%)	93 (62%)
Primary CHD diagnosis			
Tricuspid atresia	15 (15%)	7 (14%)	22 (15%)
Hypoplastic left heart syndrome	15 (15%)	7 (14%)	22 (15%)
Double inlet left ventricle	14 (14%)	9 (17%)	23 (15%)
Double outlet right ventricle	14 (14%)	9 (17%)	23 (15%)
Atrioventricular canal or atrioventricular septal defect	10 (10%)	5 (10%)	15 (10%)
Pulmonary atresia with ventricular septal defect	1 (1%)	1 (2%)	2 (1%)
Pulmonary atresia with intact ventricular septum	8 (8%)	4 (8%)	12 (8%)
Congenitally corrected transposition of the great arteries	10 (10%)	6 (12%)	16 (11%)
Other diagnosis	10 (10%)	4 (8%)	14 (9%)
Presence of syndrome or non-cardiac congenital anomaly, % yes	14 (14%)	11 (21%)	25 (17%)
Number of prior cardiac procedures prior to Fontan	2.2 ± 1.0	2.0 ± 0.8	2.1 ± 1.0
Age at Fontan operation, years	5.1 ± 2.7	5.1 ± 2.9	5.1 ± 2.8
Time since Fontan operation, years	11.6 ± 8.2	11.3 ± 8.3	11.5 ± 8.2
Fontan type			
Atrio pulmonary connection	6 (6%)	3 (6%)	9 (6%)
Lateral tunnel connection	18 (18%)	8 (15%)	26 (17%)
Extracardiac conduit	73 (74%)	40 (77%)	113 (75%)
Post-Fontan operation complications,^a^ % yes	27 (27%)	16 (31%)	43 (29%)
Cardiac complications post-Fontan, % yes	18 (18%)	7 (14%)	25 (17%)
Cardiac reinterventions post-Fontan, % yes	5 (5%)	2 (4%)	7 (5%)
Years since most recent follow-up, y	0.8 ± 0.7	0.9 ± 1.0	0.9 ± 0.8
NYHA class at follow-up			
I	32 (32%)	16 (31%)	48 (32%)
II	18 (18%)	10 (19%)	28 (19%)
Ventricular impairment at follow-up, % yes	6 (6%)	3 (6%)	9 (6%)
AV valve regurgitation at follow-up, % yes	25 (25%)	10 (19%)	35 (23%)
Parent-proxy reported child total PedsQL score	61.4 ± 19.3	64.4 ± 18.5	62.4 ± 19.0
Total time spent in intensive care	23.8 (21.1)	27.5 (34.0)	25.1 (26.4)
Total emergency admissions in past 12 months	0.7 (1.2)	0.8 (1.2)	0.7 (1.2)
Hospital admission in past 12 months, % yes	22 (22%)	10 (19%)	32 (21%)
Total length of hospital stays in past 12 months	1.7 (6.4)	3.5 (11.4)	2.3 (8.4)
Planned or surgical hospital admission(s) in next 12 months, % yes	10 (10%)	8 (15%)	18 (12%)
Total cardiology consultations in past 2 years	2.8 (2.7)	2.4 (1.0)	2.66 (2.29)
Current medication use, % yes	97 (98%)	51 (98%)	148 (98%)
Medication frequency per day	1.3 (0.9)	1.3 (0.6)	1.3 (0.8)
Challenges adhering to medication, % yes	36 (36%)	16 (31%)	52 (34%)
Advised to restrict exercise, % yes	63 (64%)	31 (60%)	94 (62%)
Dietary requirements related to cardiac condition, % yes	24 (24%)	12 (23%)	36 (24%)
Impact of dietary requirements on daily life, scale 0–4	0.6 (1.0)	0.7 (1.1)	0.6 (1.0)
Perceived seriousness of child’s heart condition, scale 0–4	3.1 (0.8)	3.0 (0.9)	3.1 (0.8)

Values are n (%) or mean ± standard deviation. Some percentages will not add to 100% owing to missing data

*CHD* congenital heart disease, *NYHA* New York Heart Association.

^a^Post-Fontan operation complications include pleural effusion chest drains in-situ > 30 days or re-operations for effusions, chylothorax < 30 days, chylothorax > 30 days, arrythmia, sepsis, mechanical support (ECMO/VAD) or other

**Fig. 1:**
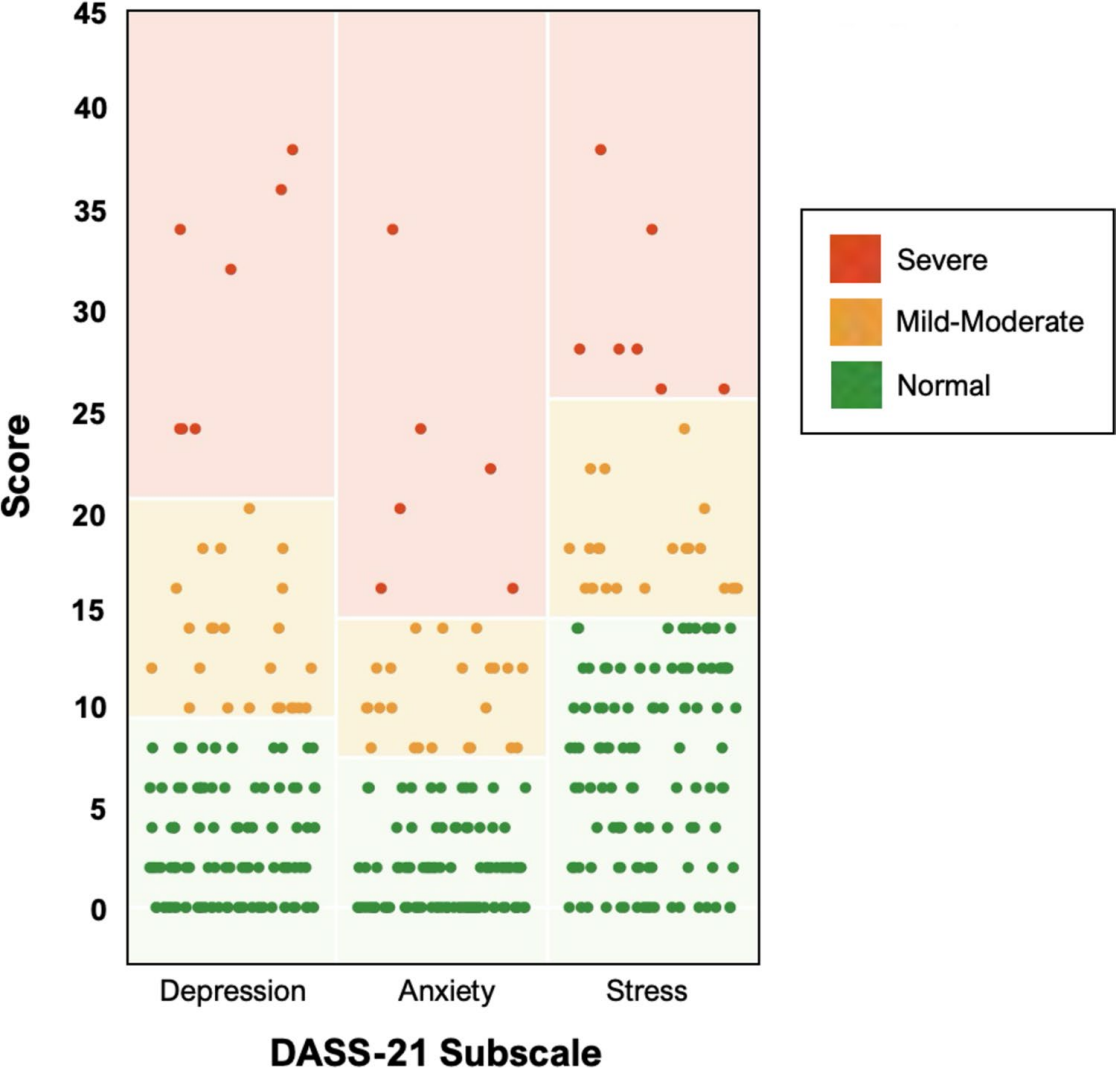
Distribution of parent DASS-21 depression, anxiety, and stress subscale scores in each clinical category

Table 2 presents the psychological, relational and health-related QOL characteristics of the sample. Parents reported higher depression, anxiety, and stress scores compared with Australian normative data [45] (all *p* < 0.001); mothers and fathers did not differ in terms of depression (*p* = 0.47), anxiety (*p* = 0.84), or stress (*p* = 0.90) scores. Thirty-one percent of parents reported scores on at least one DASS-21 subscale indicative of a need for clinical intervention, with 20, 21, and 20% scoring in the at-risk range for depression, anxiety, and stress, respectively (Fig. 1). Perceived social support was ‘high’ among parents (M = 5.6 ± 1.2). Almost all parents (98%) exceeded the GF-12 threshold (≥ 2.0), indicative of difficulties in family functioning (*M* = 3.3 ± 0.5). Half of parents (50%) reported being offered emotional support by a health professional and 21% reported challenges accessing professional support, primarily due to cost (81%), belief in self-reliance (67%), and uncertainty about where to seek support (57%). Of those who reported receiving emotional support (29%), 13% accessed this through their child’s hospital or congenital heart center, 12% sought psychological services outside their cardiac center, and 44% found the support beneficial. Most (87%) reported being ‘highly’ to ‘extremely’ satisfied with their child’s cardiac care.

**Table 2:** Parent psychological, relational and health-related QOL characteristics

Variable	Mothers (*n* = 99)	Fathers (*n* = 52)	Total Sample (*N* = 151)
*Psychological characteristics*			
Depressive symptoms	5.7 ± 7.7	6.6 ± 6.9	6.0 ± 7.4
Anxiety symptoms	3.9 ± 5.8	4.1 ± 5.0	4.0 ± 5.5
Psychological stress	9.7 ± 7.4	9.8 ± 7.8	9.7 ± 7.5
Traumatic stress symptoms	8.5 ± 11.2	7.5 ± 10.3	8.2 ± 10.9
Sense of coherence	64.7 ± 13.3	65.5 ± 12.9	65.0 ± 13.1
*Relational characteristics*			
Attachment style			
Attachment anxiety	2.8 ± 0.8	2.9 ± 0.8	2.8 ± 0.8
Attachment avoidance	3.1 ± 0.7	3.0 ± 0.6	3.1 ± 0.6
Perceived social support	5.6 ± 1.3	5.7 ± 1.1	5.6 ± 1.24
Family functioning	3.3 ± 0.5	3.3 ± 0.5	3.3 ± 0.5
Perceived impact of CHD on family	45.4 ± 8.9	47.4 ± 8.1	46.1 ± 8.6
Parental sensitivity			
Pre-mentalizing modes	2.8 ± 0.6	2.8 ± 0.7	2.8 ± 0.6
Certainty about mental states	3.9 ± 1.1	3.7 ± 0.9	3.8 ± 1.1
Interest and curiosity in mental states	5.4 ± 1.0	5.2 ± 0.8	5.3 ± 0.9
Access to emotional support			
Offered emotional support by health professional, % yes	51 (52%)	25 (48%)	76 (50%)
Difficulties accessing emotional support, % yes	26 (26%)	5 (10%)	31 (21%)
Perceived benefit of emotional support, scale 0–4	2.4 (1.2)	2.0 (1.2)	2.3 (1.2)
Satisfaction with cardiac care, scale 0–4	3.3 ± 0.9	3.4 ± 0.6	3.3 ± 0.8
*Health-related quality of life (PedsQL scores)*^a^			
Total PedsQL score	74.9 ± 15.1	78.9 ± 13.7	76.3 ± 14.8
Physical functioning	73.0 ± 21.3	80.0 ± 17.2	75.4 ± 20.2
Psychosocial functioning	76.2 ± 14.5	78.5 ± 14.4	77.0 ± 14.4
Emotional functioning	65.0 ± 19.4	71.4 ± 18.6	67.2 ± 19.3
Social functioning	86.0 ± 15.3	86.4 ± 13.0	86.1 ± 14.5
Occupational functioning	76.4 ± 19.0	77.0 ± 15.7	76.6 ± 17.9

Values are n(%) or mean ± standard deviation

*CHD* congenital heart disease

^a^Lower PedsQL scores indicate poorer HRQOL

### Health-related quality of life

**Fig. 2:**
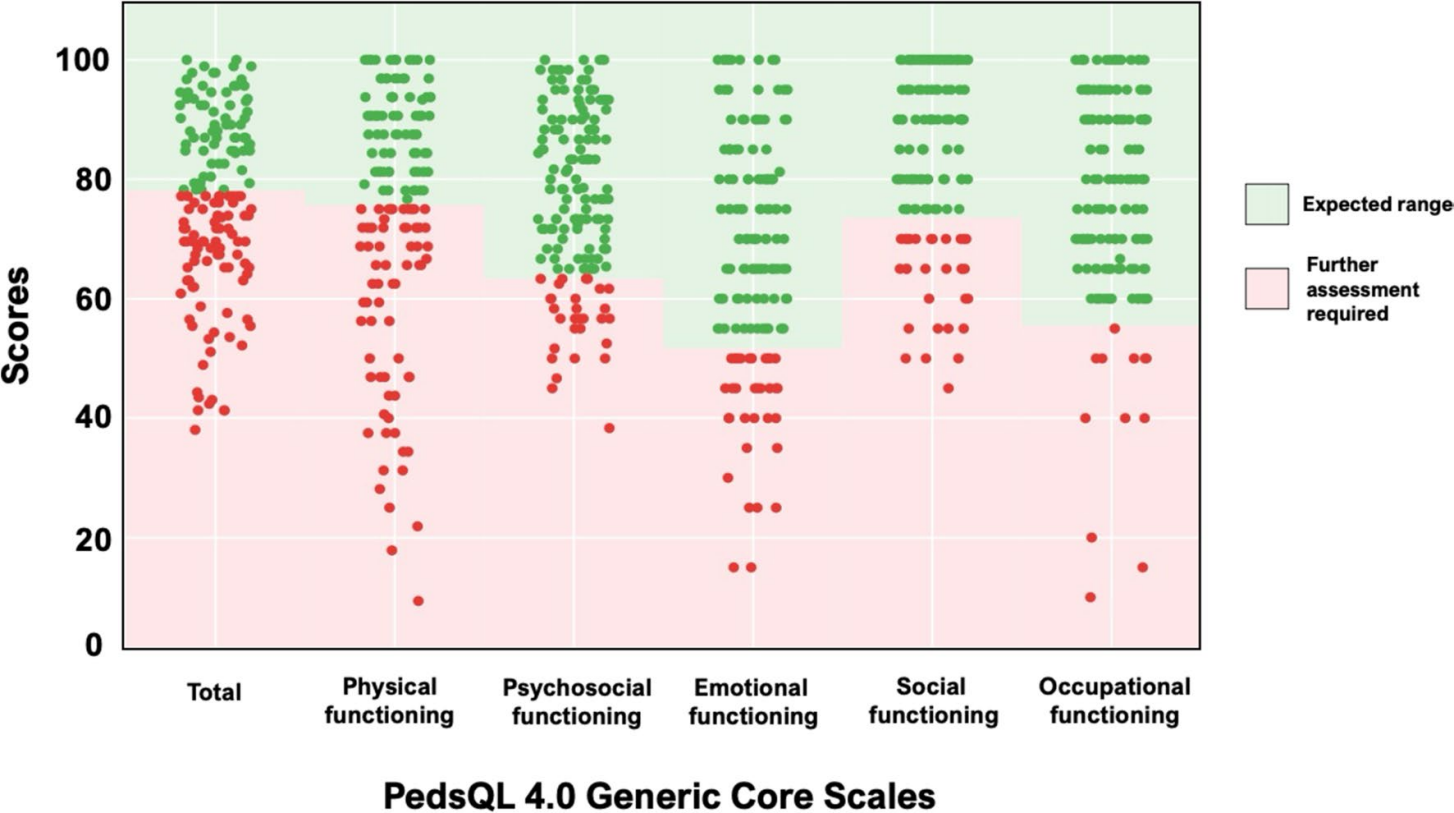
Proportion of parents of individuals with Fontan physiology enrolled in the Australian and New Zealand Fontan Registry scoring in the at-risk range of the PedsQL Core Scales

Over a quarter of parents (28%) reported total health-related QOL scores within the at-risk range (Fig. 2). Physical functioning was the most affected domain (44%), followed by emotional (24%) social (20%), psychosocial (20%) and occupational functioning (8%). Mothers reported lower physical functioning compared with fathers (*p* = 0.03), with no differences between groups in other PedsQL domains.

The final model accounted for 57% of the variance in total health-related QOL (Table 3a). Sociodemographic factors (Block 1) including age, sex, and education, initially accounted for 1% of the variance in total PedsQL scores (QIC: 29,093.84). Presence of an acute or chronic health condition (Block 2) lowered the QIC value to 26,070.21 and increased the total explained variance to 12%. Child HLHS diagnosis, emergency admissions in the past 12 months, cardiac complications post-Fontan, and exercise restrictions (Block 3, child cardiac-related factors) increased the total explained variance to 26% (QIC: 21,864.70). Psychological stress and sense of coherence (Block 4) accounted for an additional 30% of the variance, bringing the total explained variance to 56% (QIC: 12,937.48). Perceived social support (Block 5) marginally improved model fit, yielding a final QIC value of 12,693.77 and marginal *R*^2^ of 0.57.

**Table 3:** Multivariable regression model of global and health-related quality of life (total PedsQL scores) predictors, accounting for familial clustering using generalized estimating equations (*N* = 127)

(a) Health-related quality of life	Estimate (SE)	95% CIs		*p*	QIC^a^	Marginal *R*^2^
*Block 1: Sociodemographic characteristics*						
Age	− 0.22 (0.14)	− 0.50	0.05	0.11	29,476.71	0.0001
Male	2.84 (1.71)	− 0.52	6.20	0.10	29,227.10	0.01
University educated	− 3.12 (1.96)	− 6.95	0.72	0.11	29,093.84	0.01
*Block 2: Parent health characteristics*						
Acute or chronic health condition, % yes^b^	− 6.15 (2.07)	− 10.20	− 2.09	**0.003**	26,070.21	0.12
*Block 3: Child cardiac characteristics*						
HLHS diagnosis	0.38 (2.67)	− 4.85	5.61	0.89	25,491.57	0.14
Total emergency admissions in past 12 months	− 0.49 (1.06)	− 2.57	1.59	0.64	24,286.59	0.18
Cardiac complications post-Fontan, % yes^b^	− 4.85 (2.78)	− 10.31	0.60	0.08	23,544.95	0.20
Advised to restrict exercise, % yes^b^	− 5.74 (2.10)	− 9.86	− 1.62	**0.01**	21,864.70	0.26
*Block 4: Psychological characteristics*						
Psychological stress	− 0.42 (0.16)	− 0.73	− 0.12	**0.01**	16,783.02	0.41
Sense of coherence	0.53 (0.11)	0.33	0.741	** < 0.001**	12,937.48	0.56
*Block 5: Relational characteristics*						
Perceived social support	0.57 (0.94)	− 1.26	2.41	0.54	12,693.77	0.57

(b) Global quality of life in the past month	Estimate (SE)	95% CIs		*p*	QIC^a^	Marginal *R*^2^
*Block 1: Sociodemographic characteristics*						
Age	− 0.02 (1.29)	− 0.05	0.01	0.15	461.79	0.0001
Male	0.17 (0.27)	− 0.36	0.71	0.52	462.24	0.002
University educated	− 0.09 (0.26)	− 0.60	0.41	0.72	462.20	0.01
Number of children	− 0.19 (0.12)	− 0.43	0.04	0.10	449.02	0.04
Perceived financial stress, scale 0–4	− 0.35 (0.15)	− 0.65	− 0.06	**0.02**	385.07	0.18
*Block 2: Child cardiac characteristics*						
HLHS diagnosis	0.18 (0.27)	− 0.36	0.71	0.50	381.69	0.19
*Block 3: Psychological characteristics*						
Psychological stress	− 0.01 (0.02)	− 0.05	0.04	0.85	358.46	0.25
Sense of coherence	0.06 (0.01)	0.04	0.09	** < 0.001**	315.49	0.35

*p* values significant at the < 0.05 level are highlighted in bold typeface

*HLHS* hypoplastic left heart syndrome, *QIC* quasi-likelihood under the independence model criterion

^a^Decreasing QIC indicates improved model fit. An exchangeable correlation structure was used

^b^1 = Yes

### Quality of life

**Fig. 3:**
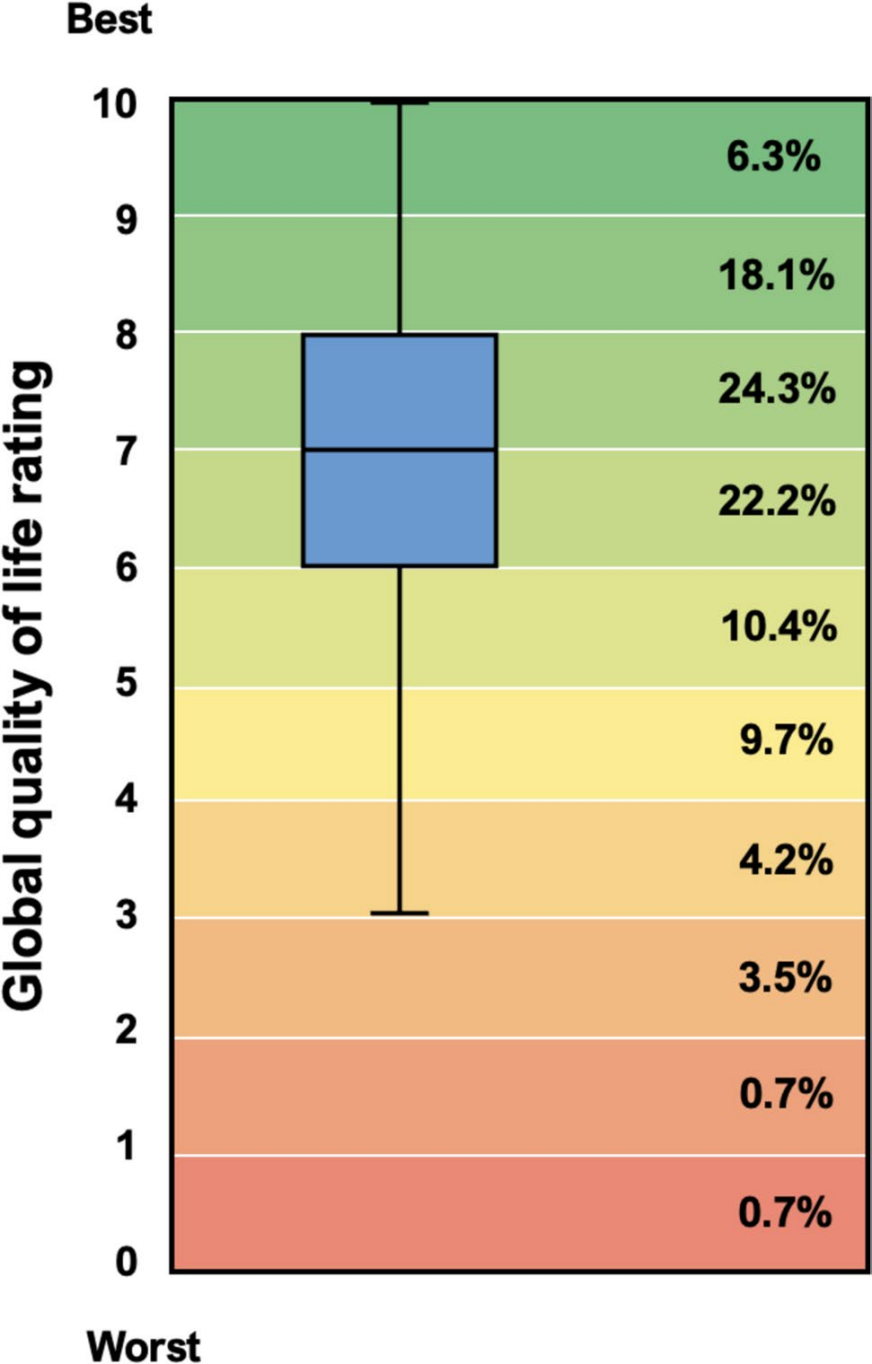
Quality of life reported by parents of children, adolescents, and adults with Fontan physiology enrolled in the Australian and New Zealand Fontan Registry. Percentages indicate the proportion of parents scoring at each level

Using a 10 cm visual analogue scale, parents reported a median global QOL score of 7.0 (IQR 6.0–8.0) over the past month, with 81% rating their QOL ≥ 6 (Fig. 3). Median scores were similar for mothers (*Median* = 7.0 [IQR 6.0–8.0]) and fathers (*Median* = 7.0 [IQR 6.0–9.0]); 79% of mothers and 86% of fathers reported QOL ≥ 6. The final model accounted for 35% of the variance in global QOL scores (Table 3b). Sociodemographic factors (Block 1), including age, sex, education, number of children in the family, and perceived financial stress accounted for 18% of the variance in global QOL scores (QIC:385.07). HLHS diagnosis (Block 2) marginally improved model fit (QIC:381.69), increasing the explained variance to 19%. Parent psychological stress and sense of coherence (Block 3) accounted for an additional 16% of the variance, yielding a final QIC value of 315.49 and marginal *R*^2^ = 0.35.

## Discussion

This study is among the first to explore global and health-related QOL among parents of individuals with Fontan physiology. Four key findings were identified. First, most parents (81%) reported good global QOL (≥ 6), consistent with broader Australian and New Zealand life satisfaction trends [57, 58]. Second, nearly one-third of parents (28%) reported at-risk health-related QOL (total PedsQL scores), with minimal gender differences across PedsQL domains. Third, psychological factors, such as psychological stress and sense of coherence, were the strongest correlates of global and health-related QOL. Fourth, in this sample, relational factors (e.g., perceived social support, family functioning) were not associated with QOL outcomes, contrary to initial hypotheses. Taken together, our findings underscore the diverse effects of caring for a child with Fontan physiology and highlight the importance of routine QOL assessment for caregivers, including measures of both global and health-related QOL, within a family-centered approach to CHD care.

Nearly half (44%) of parents reported physical functioning scores indicative of a need for further evaluation. Higher rates of chronic illness among parents of children with chronic conditions [59] may at least partially explain the physical challenges reported, with 42% of parents in our sample reporting their own acute or chronic health condition. Mothers reported lower physical functioning scores than fathers, consistent with prior CHD studies [11, 60]. For some mothers, this may be linked to increased caregiving demands which may limit personal health behaviors [61], potentially affecting physical functioning. While the prevalence of psychosocial difficulties warranting intervention (20%) aligns with prior findings [11], an additional 27% of parents scored below average, suggesting the presence of mild or unrecognized challenges that could heighten vulnerability to future stressors. Contrary to evidence indicating greater psychological distress among mothers compared with fathers [4], we found no gender differences in the psychosocial aspects of health-related QOL. This could be attributed to similarities in personal resources (e.g., perceived social support) across genders in our sample. The high proportion of couples (i.e., parents of the same child) who participated in the study (54%) could also have contributed to this finding, with evidence showing similar psychosocial functioning within parental dyads in CHD contexts [62]. Our findings reiterate the need to strengthen efforts to include fathers in CHD research.

Psychological stress and sense of coherence were the strongest correlates of global and health-related QOL, supporting our hypothesis and expanding evidence on the relative contribution of these factors [8, 10, 19] beyond the post-operative period. Parents reported greater psychological stress, anxiety, and depressive symptoms compared with the general population, with nearly one-third (31%) reporting symptoms warranting clinical evaluation, reflecting the known psychological challenges of caring for a child with complex CHD [4]. While the link between psychological stress and QOL aligns with previous evidence [8, 10, 19], our findings demonstrate the enduring effects of stress on parent wellbeing. Sense of coherence, which reflects a person’s view of life and capacity to respond to stressful situations, is associated with greater adult QOL [63], as our data also confirms. A strong sense of coherence enables individuals to understand their circumstances, use resources effectively, and find meaning in challenges, ultimately improving coping and adjustment [63, 64]. Psychological interventions, such as parent–child psychotherapy have been shown to enhance maternal psychological adjustment and wellbeing [65], and may potentially improve QOL among parents of children with Fontan physiology.

Despite relatively high perceived social support and family functioning difficulties, we found minimal association between relational factors and QOL outcomes when considered alongside psychological stress and sense of coherence, contrasting with existing evidence [8, 10, 19]. This discrepancy may stem from research focusing on the acute phases of CHD treatment (e.g., the early peri-operative period) [8, 10, 19], when social support is critical [66, 67]. Qualitative evidence shows persistent stress and health-related worry among parents as their child with Fontan physiology transitions into adolescence and adulthood [68]. Our results suggest strong social support may not fully protect parents’ QOL over time, while chronic stress may further undermine benefits of social support. Conversely, a strong sense of coherence appears key in facilitating coping with ongoing uncertainty, highlighting the importance of therapeutic approaches that evolve alongside parents’ changing needs and circumstances.

This registry-based, conceptually-driven study advances understanding of QOL among parents of individuals with Fontan physiology. A key strength is the identification of factors that significantly influence QOL, aligning with the revised Wilson and Cleary health-related QOL model [26, 27]. Other strengths are inclusion of fathers, and use of standardized outcome measures for broader comparability. Despite extensive review, we found the only available PedsQL normative data from United States [30] and Dutch adult populations [69] was unsuitable for statistical comparisons (aged ≤ 30 years), as the youngest parent in our sample was 31.6 years. We still assessed at-risk status using the most relevant available PedsQL normative data [30], which may have limited precision; however, the strong convergent validity between the PedsQL and the 36-item Short Form Health Survey (SF-36) [70] supports its accuracy in assessing adult health-related QOL. There is an urgent need for more comprehensive adult normative data for the PedsQL.

Other limitations, such as a low response rate and over-representation of English-speaking families engaged with cardiac follow-up, introduce potential selection bias and limit generalizability. Australia and New Zealand’s universal healthcare systems may affect parental QOL outcomes, limiting the applicability of results to other health systems. Evolving surgical and medical management of people born with single-ventricle CHD may also have influenced parental experiences and QOL outcomes. While we explained a considerable proportion of the variance in QOL outcomes, unexplored predictors and other mediating variables likely exist, warranting further investigation. Our sample primarily included parents of younger children (e.g., 44% of children with Fontan physiology in this study were aged ≤ 11 years), indicating a need for targeted efforts to include families of adolescents and adults in future research. Cross-sectional design limits causal inference. Data were also collected prior to the COVID-19 pandemic and may not fully reflect pandemic-related impacts on current QOL.

Examining both global and health-related QOL offers distinct insights into avenues to promote parent wellbeing. Future research could include longitudinal studies to track changes in QOL over time, alongside clinical advances and developmental transitions. Although fathers were well-represented here, they remain underrepresented in CHD research, along with other caregivers, such as grandparents, whose QOL outcomes warrant further investigation.. Regular global and health-related QOL screening is recommended for parents within pediatric and adult CHD settings. Integration of specialized psychological services within family-centered models of congenital heart care is endorsed by the American Heart Association [71] and the Australian National Standards of Care for Childhood-onset Heart Disease [72]. Embedding mental health professionals within pediatric and adult CHD services, along with routine psychosocial screening, assessment, and treatment options is likely to improve health outcomes in this population [73]. Overall, our findings highlight the resilience of parents of individuals with Fontan physiology, with most reporting satisfactory global QOL, aligning with broader societal trends. However, we also identified a substantial subset of parents reporting challenges in health-related QOL, with psychological stress and sense of coherence identified as key correlates.

## Supplementary Information

Below is the link to the electronic supplementary material.

Supplementary file1 (DOCX 133 KB)

## Data Availability

De-identified data that underlie the results reported in this article may be available to suitably qualified researchers on request, after demonstration that the proposed use of the data has been approved by an independent review committee. Data requests should be sent in writing to the corresponding author.

## Abbreviations

ANZFR: Australian and New Zealand Fontan Registry
CHD: Congenital heart disease
HLHS: Hypoplastic left heart syndrome
HRQOL: Health-related quality of life
QOL: Quality of life
