# Supplementary material for: Wellbeing and quality of life among parents of individuals with Fontan physiology
Source: Qual Life Res. 2025 Jan 22;34(5):1419–31. doi: 10.1007/s11136-025-03890-6 (PMC12064451; doi:10.1007/s11136-025-03890-6)
Supplement: Supplementary file 1 — Supplementary file1 (DOCX 133 KB) [file 11136_2025_3890_MOESM1_ESM.docx]

**SUPPLEMENTARY INFORMATION**

**Supplementary table 1. Sociodemographic, clinical, and psychological of quality of life among parents of individuals with Fontan physiology.**

|  | **Health-related quality of life** | | | | **Global quality of life** | | | |
| --- | --- | --- | --- | --- | --- | --- | --- | --- |
| **Variable** | **Estimate (SE)** | ***p*** | ***95% CI*** | | **Estimate (SE)** | ***p*** | ***95% CI*** | |
| ***Sociodemographic factors*** |  |  |  |  |  |  |  |  |
| Age at assessment, years | -0.03 (0.11) | 0.79 | -0.26 | 0.20 | -0.01 (0.01) | 0.07 | -0.03 | 0.02 |
| Sex |  |  |  |  |  |  |  |  |
| Female | * |  |  |  | * |  |  |  |
| Male | 4.02 (2.51) | 0.12 | -0.94 | 8.98 | 0.20 (0.32) | 0.53 | -0.43 | 0.84 |
| Country of birth |  |  |  |  |  |  |  |  |
| Australia | * |  |  |  | * |  |  |  |
| New Zealand | 3.27 (3.73) | 0.38 | -4.10 | 10.64 | 0.09 (0.46) | 0.84 | -0.83 | 1.01 |
| Other | 0.74 (3.07) | 0.81 | -5.31 | 6.80 | -0.02 (0.39) | 0.96 | -0.78 | 0.75 |
| Language primarily spoken at home |  |  |  |  |  |  |  |  |
| English | * |  |  |  | * |  |  |  |
| Other | -0.29 (6.23) | 0.96 | -12.59 | 12.01 | 0.04(0.77) | 0.96 | -1.48 | 1.57 |
| Marital status |  |  |  |  |  |  |  |  |
| Not married | * |  |  |  | * |  |  |  |
| Married or partnered | 1.58 (4.72) | 0.74 | -7.75 | 10.91 | 0.54 (0.61) | 0.37 | -0.66 | 1.74 |
| Number of children | -1.55 (1.17) | 0.18 | -3.86 | 0.75 | -0.32 (0.15) | **0.03** | -0.61 | -0.02 |
| Fontan patient first-born or only child, % yes | 1.30 (2.58) | 0.62 | -3.80 | 6.39 | 0.43 (0.31) | 1.77 | -0.18 | 1.06 |
| Educational attainment |  |  |  |  |  |  |  |  |
| No university degree | * |  |  |  | * |  |  |  |
| University degree | -1.06 (2.44) | 0.66 | -6.36 | 3.28 | -0.34 (0.31) | 0.27 | -0.95 | 0.27 |
| Employment status |  |  |  |  |  |  |  |  |
| Employed (e.g., full-, part-time, casual) | * |  |  |  | * |  |  |  |
| Unemployed (incl. job-seeking, disability pension) | -15.11 (5.25) | **0.005** | -25.49 | -4.72 | -0.63 (0.68) | 0.35 | -1.96 | 0.71 |
| Unemployed, not looking for work (e.g., retired, carer) | -5.85 (3.07) | 0.06 | -11.02 | 0.23 | -0.48 (0.40) | 0.23 | -1.28 | 0.31 |
| Employed hours per week, hours | 0.02 (0.02) | 0.27 | -0.02 | 0.07 | 0.004 (0.003) | 0.90 | -0.01 | 0.01 |
| Gross weekly household income |  |  |  |  |  |  |  |  |
| Below national average | * |  |  |  | * |  |  |  |
| Above national average | 6.32 (2.82) | **0.02** | 0.73 | 11.92 | 0.66 (0.36) | 0.07 | -0.05 | 1.37 |
| Perceived financial stress, scale 0-4 | -6.27 (1.05) | **<0.001** | -8.36 | -4.18 | -0.70 (0.14) | **<0.001** | -0.97 | -0.43 |
| Residential location |  |  |  |  |  |  |  |  |
| Metropolitan or urban | * |  |  |  | * |  |  |  |
| Regional or rural | -2.20 (2.94) | 0.45 | -8.01 | 3.62 | 0.20 (0.38) | 0.59 | -0.54 | 0.95 |
| Health literacy, scale 0-8 | 3.00 (1.14) | **0.01** | 0.75 | 5.25 | 0.15 (0.14) | 0.31 | -0.14 | 0.44 |
| ***Parent health characteristics*** |  |  |  |  |  |  |  |  |
| Presence of comorbidities, % yes | -7.71 (2.34) | **0.003** | -12.35 | -3.08 | -0.04(0.31) | 0.91 | -0.65 | 0.58 |
| Medication use, % yes | -8.15 (2.35) | **0.001** | -12.80 | -3.50 | -0.28 (0.31) | 0.35 | -0.90 | 0.32 |
| ***Child cardiac characteristics*** |  |  |  |  |  |  |  |  |
| Child age, years | 0.11 (0.13) | 0.42 | -0.16 | 0.38 | 0.01 (0.02) | 0.54 | -0.02 | 0.04 |
| Child sex |  |  |  |  |  |  |  |  |
| Female | * |  |  |  | * |  |  |  |
| Male | 4.82 (2.42) | **0.04** | 0.04 | 9.60 | 0.02 (0.31) | 0.96 | -0.61 | 0.63 |
| Prenatal diagnosis, % yes | 0.24 (2.48) | 0.92 | -4.67 | 5.12 | -0.01 (0.32) | 0.98 | -0.63 | 0.61 |
| HLHS diagnosis, % yes | -7.43 (3.35) | **0.03** | -14.06 | -0.79 | -0.89 (0.42) | **0.04** | -1.72 | -0.06 |
| Presence of syndrome or non-cardiac congenital anomaly, % yes | -0.04 (3.31) | 0.99 | -6.60 | 6.51 | 0.20 (0.43) | 0.63 | -0.63 | 1.05 |
| Number of prior cardiac procedures prior to Fontan | 0.18 (1.31) | 0.89 | -2.42 | 2.77 | -0.13 (0.17) | 0.46 | -0.46 | 0.20 |
| Age at Fontan operation, years | 0.47 (0.44) | 0.29 | -0.41 | 1.35 | 0.03 (0.05) | 0.58 | -0.09 | 0.15 |
| Time since Fontan operation, years | 0.03 (0.15) | 0.83 | -0.26 | 0.33 | 0.01 (0.02) | 0.55 | -0.02 | 0.05 |
| Fontan type |  |  |  |  |  |  |  |  |
| Atrio pulmonary connection | * |  |  |  | * |  |  |  |
| Lateral tunnel connection | -2.48 (3.18) | 0.44 | -8.76 | 3.81 | 0.21 (0.41) | 0.59 | -0.60 | 1.03 |
| Extracardiac conduit | 1.92 (2.77) | 0.49 | -3.55 | 7.39 | -0.22 (0.36) | 0.54 | -0.94 | 0.49 |
| Post-Fontan operation complications, % yes | 0.61 (2.69) | 0.82 | -4.71 | 5.92 | 0.13 (0.34) | 0.70 | -0.54 | 0.80 |
| Cardiac complications post-Fontan, % yes | -6.90 (3.18) | **0.03** | -13.20 | -0.60 | -0.34 (0.43) | 0.42 | -1.18 | 0.50 |
| Cardiac reinterventions, % yes | -9.43 (5.677 | 0.09 | -20.65 | 1.77 | -1.44 (0.92) | 0.12 | -3.27 | 0.39 |
| Years since most recent follow-up, y | 0.87 (1.91) | 0.65 | -2.91 | 4.65 | 0.87 (1.90) | 0.65 | -2.91 | 4.65 |
| NYHA class at follow-up |  |  |  |  |  |  |  |  |
| I | * |  |  |  | * |  |  |  |
| II | -6.03 (3.57) | 0.10 | -13.15 | 1.09 | -0.44 (0.44) | 0.31 | 1.31 | 0.42 |
| Ventricular impairment at follow-up, % yes | 2.72 (5.08) | 0.59 | -7.31 | 12.77 | 1.14 (0.62) | 0.07 | -0.10 | 2.38 |
| AV valve regurgitation at follow-up, % yes | -3.99 (2.83) | 0.16 | -9.59 | 1.61 | -0.56 (0.37) | 0.13 | -1.28 | 0.17 |
| Parent-proxy reported child total PedsQL score | 0.35 (0.06) | **<0.001** | 0.24 | 0.46 | 0.01 (0.01) | 0.14 | -0.004 | 0.03 |
| Total time spent in intensive care | -0.02 (0.05) | 0.59 | -0.11 | 0.07 | 0.002 (0.01) | 0.70 | -0.01 | 0.01 |
| Total emergency admissions in past 12 months | -2.48 (1.01) | 0.02 | -4.48 | -0.48 | 0.05 (0.13) | 0.69 | -0.20 | 0.31 |
| Hospital admission in past 12 months, % yes | -3.15 (2.95) | 0.27 | -8.99 | 2.68 | 0.18 (0.38) | 0.64 | -0.58 | 0.93 |
| Total length of hospital stay in past 12 months | -0.21 (0.15) | 0.17 | -0.52 | 0.10 | 0.001 (0.02) | 0.93 | -0.04 | 0.04 |
| Planned or surgical hospital admission(s) in next 12 months, % yes | -2.09 (3.71) | 0.57 | -9.43 | 5.25 | 0.27 (0.46) | 0.56 | -0.63 | 1.19 |
| Total cardiology consultations in past 2 years | -0.42 (0.53) | 0.42 | -1.47 | 0.63 | -0.03 (0.07) | 6.10 | -0.17 | 0.10 |
| Current medication use, % yes | -13.83 (8.55) | 0.11 | -30.73 | 3.07 | -1.21 (1.07) | 0.26 | -3.32 | 0.91 |
| Medication frequency per day | -1.42 (1.58) | 0.37 | -4.56 | 1.70 | -0.12 (0.20 | 0.55 | -0.52 | 0.28 |
| Challenges adhering to medication, % yes | 1.16 (2.53) | 0.65 | -3.85 | 6.16 | -0.93 (0.31) | 0.003 | -1.54 | -0.31 |
| Advised to restrict exercise, % yes | -9.65 (2.40) | **<0.001** | -14.41 | -4.90 | -0.70 (0.32) | 0.03 | -1.33 | -0.06 |
| Dietary requirements related to cardiac condition, % yes | -0.87 (2.83) | 0.75 | -6.47 | 4.73 | 0.05 (0.37) | 0.89 | -0.69 | 0.79 |
| Impact of dietary requirements on daily life, scale 0-4 | -0.04 (0.03) | 0.19 | -0.09 | 0.02 | -0.002 (0.003) | 0.537 | -0.009 | 0.004 |
| Perceived seriousness of child’s heart condition, scale 0-4 | -0.13 (1.44) | 0.93 | -2.99 | 2.72 | 0.15 (0.18) | 0.38 | -0.20 | 0.51 |
| ***Psychological characteristics*** |  |  |  |  |  |  |  |  |
| Depressive symptoms | -1.19 (0.14) | **<0.001** | -1.46 | -0.91 | -0.13 (0.01) | **<0.001** | -0.16 | -0.09 |
| Anxiety symptoms | -1.40 (0.12) | **<0.001** | -1.80 | -1.01 | -0.12 (0.03) | **<0.001** | -0.17 | -0.07 |
| Psychological stress | -1.05 (0.15) | **<0.001** | -1.34 | -0.76 | -0.09 (0.02) | **<0.001** | -0.13 | -0.05 |
| Traumatic stress symptoms | -0.45 (0.11) | **0.001** | -0.66 | -0.23 | -0.03 (0.01) | **0.02** | -0.06 | -0.01 |
| Sense of coherence | 0.68 (0.08) | **<0.001** | 0.54 | 0.83 | 0.08 (0.01) | **<0.001** | 0.06 | 0.10 |
| ***Relational characteristics*** |  |  |  |  |  |  |  |  |
| Attachment style |  |  |  |  |  |  |  |  |
| Attachment anxiety | -9.44 (1.40) | **<0.001** | -12.22 | -6.67 | -1.08 (0.23) | **<0.001** | -1.43 | -0.72 |
| Attachment avoidance | -8.94 (1.79) | **<0.001** | -12.48 | -5.40 | -0.70 (0.18) | **0.003** | -1.16 | -0.23 |
| Perceived social support | 3.53 (0.97) | **<0.001** | -2.76 | 3.21 | 0.31 (0.12) | **0.01** | 0.07 | 0.55 |
| Family functioning | 12.45 (2.30) | **<0.001** | 7.90 | 17.00 | 0.87 (0.32) | **0.01** | 0.24 | 1.49 |
| Perceived impact of CHD on family | 0.76 (0.14) | **<0.001** | 0.48 | 1.04 | 0.05 (0.02) | **0.01** | 0.01 | 0.09 |
| Parental sensitivity |  |  |  |  |  |  |  |  |
| Pre-mentalizing modes | -0.77 (2.21) | 0.73 | -5.16 | 3.62 | 0.01 (0.29) | 0.97 | -0.56 | 0.59 |
| Certainty about mental states | 1.57 (1.33) | 0.24 | -1.06 | 4.20 | 0.19 (0.17) | 0.27 | -0.15 | 0.53 |
| Interest and curiosity in mental states | 0.23 (1.50) | 0.88 | -2.76 | 3.21 | -0.01 (0.20) | 0.96 | -0.40 | 0.38 |
| Access to emotional support |  |  |  |  |  |  |  |  |
| Offered emotional support by health professional, % yes | -2.58 (2.50) | 0.30 | -7.53 | 2.35 | -0.20 (0.31) | 0.51 | -0.81 | 0.41 |
| Difficulties accessing emotional support, % yes | -12.38 (2.88) | **<0.001** | -18.08 | -6.67 | -0.73 (0.37) | 0.05 | -1.47 | 0.00 |
| Perceived benefit of emotional support, scale 0-4 | 3.07 (1.36) | **0.03** | 0.34 | 5.79 | 0.36 (0.16) | **0.03** | 0.03 | 0.69 |
| Satisfaction with cardiac care, scale 0-4 | 4.99 (1.47) | **0.001** | 2.09 | 7.89 | 0.32 (0.19) | 0.09 | -0.05 | 0.69 |

CHD, congenital heart disease; HLHS, hypoplastic left heart syndrome. NYHA, New York Heart Association. *p* values significant at the <.05 level are highlighted in bold typeface. *Reference category.

**
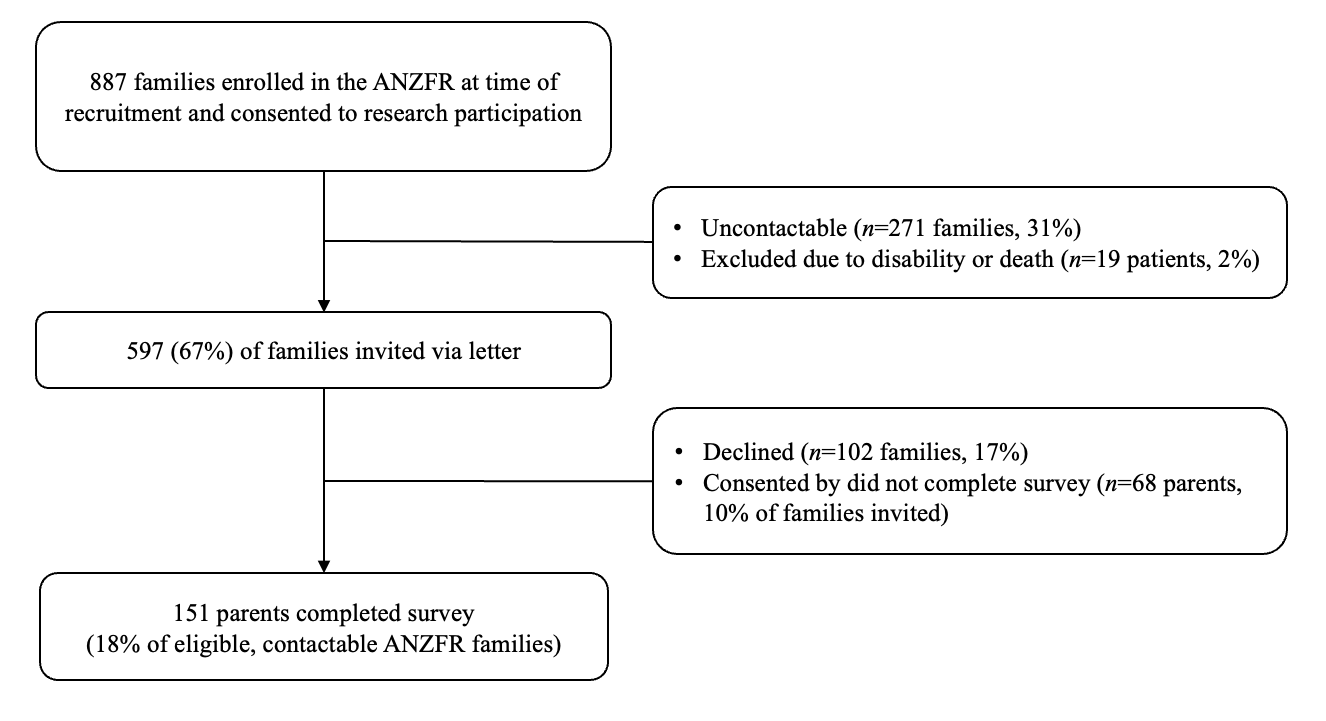
**

**Supplementary Figure 1. Flowchart of participant recruitment.**
